# First Diagnosed Case of Camelpox Virus in Israel

**DOI:** 10.3390/v10020078

**Published:** 2018-02-13

**Authors:** Oran Erster, Sharon Melamed, Nir Paran, Shay Weiss, Yevgeny Khinich, Boris Gelman, Aharon Solomony, Orly Laskar-Levy

**Affiliations:** 1 Division of Virology, Kimron Veterinary Institute, P.O. Box 12, Beit Dagan 50250, Israel; Orane@moag.gov.il (O.E.); vaccine@moag.gov.il (Y.K.); Borisg@moag.gov.il (B.G); 2 Department of Infectious Diseases, IIBR P.O. Box 19, Ness Ziona 74100, Israel; sharonm@iibr.gov.il (S.M.); nirp@iibr.gov.il (N.P.); shayw@iibr.gov.il (S.W.); 3 Negev Veterinary Bureau, Israeli Veterinary Services, Binyamin Ben Asa 1, Be′er Sheba 84102, Israel; Aharons@moag.gov.il

**Keywords:** *orthopoxvirus*, camelpox virus, transmission electron microscopy, PCR, Immunofluorescence assay (IFA), chorioallantoic membrane

## Abstract

An outbreak of a disease in camels with skin lesions was reported in Israel during 2016. To identify the etiological agent of this illness, we employed a multidisciplinary diagnostic approach. Transmission electron microscopy (TEM) analysis of lesion material revealed the presence of an orthopox-like virus, based on its characteristic brick shape. The virus from the skin lesions successfully infected chorioallantoic membranes and induced cytopathic effect in Vero cells, which were subsequently positively stained by an orthopox-specific antibody. The definite identification of the virus was accomplished by two independent qPCR, one of which was developed in this study, followed by sequencing of several regions of the viral genome. The qPCR and sequencing results confirmed the presence of camelpox virus (CMLV), and indicated that it is different from the previously annotated CMLV sequence available from GenBank. This is the first reported case of CMLV in Israel, and the first description of the isolated CMLV subtype.

## 1. Introduction

Pox and pox-like diseases belongs to a group of systemic diseases that in their classical form are manifested by skin lesions with various morphologies [1]. In camels, the diagnosis of such skin lesions involves three main suspected viruses that can cause similar skin lesions [2]. camelpox virus (CMLV), the causative agent of camelpox, belongs to the genus *Orthopoxvirus* (OPV), and Camel contagious ecthyma virus (CCEV), the causative agent of Auzdik disease, which belongs to the genus *Parapoxvirus* (PPV). The third virus is camelus dromedary papillomavirus, the causative agent of camel papillomatosis [3]. Although the clinical signs may look similar, the consequences of each viral infection are completely different in terms of possible human infection [4,5] and economic implication [6,7].

Phylogenetic analysis shows that among OPV, CMLV is the closest to variola virus (VARV), the causative agent of smallpox [8]. Despite the above, each virus exhibits a strictly narrow host range [9]; VARV exhibits exclusive human-specificity, whereas CMLV infect exclusively old world camelids [7], with very rare human infection cases [5].

Camel contagious ecthyma virus [10] (CCEV) induces acute cutaneous pustular lesions in camels that can be transmitted to humans. In humans, the lesions remain localized, and infections on the hand are relatively common among people working in close contact with animals. It was previously shown that orf virus, the prototype of PPV, has adapted to skin tissue via unique genes that were acquired from the host during evolution [11,12].

Camelus dromedary papillomavirus (CDPV) belongs to the genus Delta papillomavirus of the family Papillomaviridae [3], which can be clearly distinguished using Transmission Electron Microscopy (TEM), as its shape and size is completely different from that of poxviruses.

CMLV is endemic in Africa and Asia, circulating in old-world camelids [13]. Camels are used as a source of wool, milk, and meat. Therefore, the emergence of such a disease is of economic importance, with implications on animal handlers’ health.

Here, we describe the first diagnosed cases of CMLV in Israel using a sensitive, specific, and rapid diagnostic approach for differentiating between CMLV, CCEV, and CDPV. The first method includes imaging the infectious agent using TEM. This rapid method enables the differentiation among papillomavirus, OPV, and PPV. In case of insufficient material, an isolation of virus particles can be achieved by infecting chorioallantoic membranes or cell cultures. As supporting evidence for the presence of OPV, we performed an immunofluorescence assay on infected cells using an orthopox-specific antibody. Pock formation on chorioallantoic membranes, and the induction of typical cytopathic effects, are common accessory methods that are used in parallel. Lastly, a combination of PCR and DNA sequencing approaches were used for the definite identification of CMLV.

## 2. Materials and Methods

### 2.1. Cells and Viruses

BS-C-1 (ATCC, CCL-26) and Vero (ATCC CCL-81) cells were routinely maintained in Dulbecco′s modified Eagle medium (DMEM) supplemented with 10% fetal calf serum, 2 mM of glutamine, 0.1 mg/mL of streptomycin, 100 units/mL of penicillin, 1.25 units/mL of nystatin, and non-essential amino acids (Biological Industries, Beit-Haemek, Israel). Vaccinia virus-Western Reserve (VACV-WR, ATCC VR-119) and cowpox virus (CPXV, strain Brighton, ATCC VR-302) were grown in HeLa cells, and purified as described previously [14]. CMLV titers were determined by plaque assay on Vero cells, as described elsewhere [15].

### 2.2. Samples and Antibodies

Samples from eight animals showing clinical signs that are characteristic of a poxvirus infection, were collected from four locations in the Israel desert (Negev) and southeastern areas as follows: two samples from Hura, two from Kseifeh, one from Tel Arad, and three from the Hebron region. All of the diagnostic assays in this study were performed on skin scrapes, except qPCR, which was also performed on whole blood samples. Preparation of the anti-VACV hyperimmune serum was described previously [16,17].

### 2.3. TEM Imaging

A small piece of skin lesion was processed by vortexing in PBS, then inactivation and fixation were performed using 2% buffered formaldehyde (FA) for 30 min at 25 °C, followed by an additional 30 min of incubation at 25 °C [18]. A volume of 10 μL was applied on 1% Alcian blue treated, 300 mesh carbon-coated copper TEM grids. Samples were incubated for 10 min, and then excess liquid was blotted using Whatman paper. Grids were washed three times with distilled water, and stained with 1% phosphotungstic acid. After air-drying, samples were visualized using an Tecnai T12 TEM (Thermo Fisher, Oregon, USA) operated at 120 kV and equipped with a Gatan ES500W Erlangshen camera. Scaling was done using a standard of known size measured at different magnifications. The viral length of each particle was measured by stretching a line from end to end.

### 2.4. Infection of Chorioalanthoic Membrane & Vero Cells

In order to isolate the virus, lysates from skin samples were used to inoculate specific pathogen free embryonated chicken eggs (SPF-ECE) and Vero cells. Skin samples from suspected camels were ground in sterile PBS supplemented with 0.1 mg/mL of streptomycin, 100 units/mL of penicillin, and 1.25 units/mL of nystatin antibiotic mix. The extract was layered on chorioallantoic membranes (CAM) of 10-days old SPF-ECE using a 23 G sterile needle. Following egg death, the chorioallantoic membrane was excised and used to generate an infectious suspension for Vero cells infection. Vero cells were infected with the SPF-ECE CAM resuspension, and grown under standard conditions.

### 2.5. Infection, Immunofluorescence Assay (IFA) and Cells Staining

For immunofluorescence labeling, Vero cells were plated on LabTek (Thermo Scientific Nunc, Waltham, MA, USA). Following the different infections and treatments, cells were fixed with 3% paraformaldehyde (PFA) in PBS for 20 min, and permeabilized with 0.5% Triton X-100 for 2 min. The fixed cells were rinsed with PBS and blocked with PBS supplemented with 2% Bovine Serum Albumin (Sigma, St. Louis, MI, USA), and the LabTek were incubated for 45 min with anti-VACV hyperimmune serum. Following the PBS washes, the cells were incubated with the appropriate fluorescently conjugated secondary antibodies for 30 min. For nuclei visualization, cells were stained with 5 μg/mL (4′,6-diamidino-2-phenylindole) DAPI [17].

For cytopathic effect and syncytial formation, cells were infected (Figure 4) and fixed with 3.7% formalin for 20 min. Hematoxylin-eosin staining was performed using the EnVision kit (Dakocytomation, Carpinteria, CA, USA). Light and fluorescence microscopy were carried out using the Nikon TE2000 fluorescent microscope (Tokyo, Japan). The images were acquired by a Nikon DXM-1200F camera, and processed with Adobe Photoshop software (5.0, San Jose, CA, USA).

### 2.6. Primer Design and Multiple Alignment Analysis

Primers were designed and tested in silico using the Geneious software (Biomatters, version 8.0, Auckland, New Zealand). A multiple alignment analysis of partial genomic regions was performed using the MUSCLE algorithm [19]. Alignments of complete Poxvirus genomes were performed using the BLASTZ algorithm [20]. The alignment file was then used to construct a phylogenetic tree for the tested region, using the Mega6 software [21], according to the maximum likelihood method.

### 2.7. DNA Extraction and PCR Procedures

DNA was extracted from blood and skin samples using the iNTRON viral extraction kit (http://intronbio.com/eng/viral-gene-spin.html). Two quantitative PCR (qPCR) assays were used. The first one was based on the C18L gene described by Balamurugan [22], using the following primers: C18L_F5′-GCGTTAACGCGACGTCGTG-3′ and C18L_R5′-GATCGGAGATATC ATACTTT ACTTTAG-3′. The reaction mix was prepared as follows: 2 μL of DNA template, 5 μL of PerfeCTa SYBR^®^ Green SuperMix (http://www.quantabio.com/perfecta-syber-green-supermix), and 0.4 μM each of primer and ddH_2_O, to a final reaction volume of 10 μL. Reaction conditions were: 95 °C 5′, [95 °C 10′, 62–64.4 °C 30′ (plate read)] × 40, melt curve: 70.0 °C to 84.4 °C, increment of 0.4 °C for 1 min. A second PCR, termed 137637, was designed during the course of this study, and directed to amplify a different region in the CMLV genome. Two primers were designed to target a 192 bp region located downstream to the gene encoding RNA polymerase subunit RPO132 (acc. No. NC_003391). This region is approximately 61Kb upstream to the C18L region. The primers sequence was as follows: CMLV_137637_FWD 5′-AGTTGATCTCAAGCCGTC-3′, and CMLV_137829_REV 5′-GAGGTGTAACAGCCACTTG-3′. The reaction mix and conditions were as described for the C18L reaction. The sensitivity of the test was assayed using decimal dilutions of a purified control PCR product that contained the reaction target sequence. The primers that were used to generate the control PCR product were as follows: CMLV_137401F 5′-GTTTTAAAAGACTCAGAAGAGG-3′, and CMLV_138164R 5′-AACGTGAATTGGAATCTGAACG-3′. The conditions for the control product amplification were as described for the standard PCR run above.

In order to characterize the infecting virus, three regions of the genomes were amplified and sequenced. The primer sets designed for the characterization procedure are listed in Table 1.

The mix for each reaction was assembled as follows: 10 μL of Takara EmeraldAmp mix (http://ww.clontech.com/), 2–4 μL of the DNA extraction, 0.4 μM of the final concentration of each primer, and ddH_2_O to a final reaction volume of 20 μL. The following conditions were used in a ProFlex thermocycler (Thermo Fisher, Waltham, MA, USA): 98 °C 1′, (98 °C 20″, 51 °C 50″, 72 °C 20″) x 35, 72 °C 5′. PCR products were resolved on agarose 1.2% gel, and visualized using Olerup SSP GelReddye (http://www.olerup.com/). For the identification of CPXV and VACV DNA, the extension time was extended to 1′25″. The PCR products were purified either following gel separation or directly from the reaction tube, and were sequenced.

## 3. Results

### 3.1. Case Description and Clinical Signs

**Figure 1:**
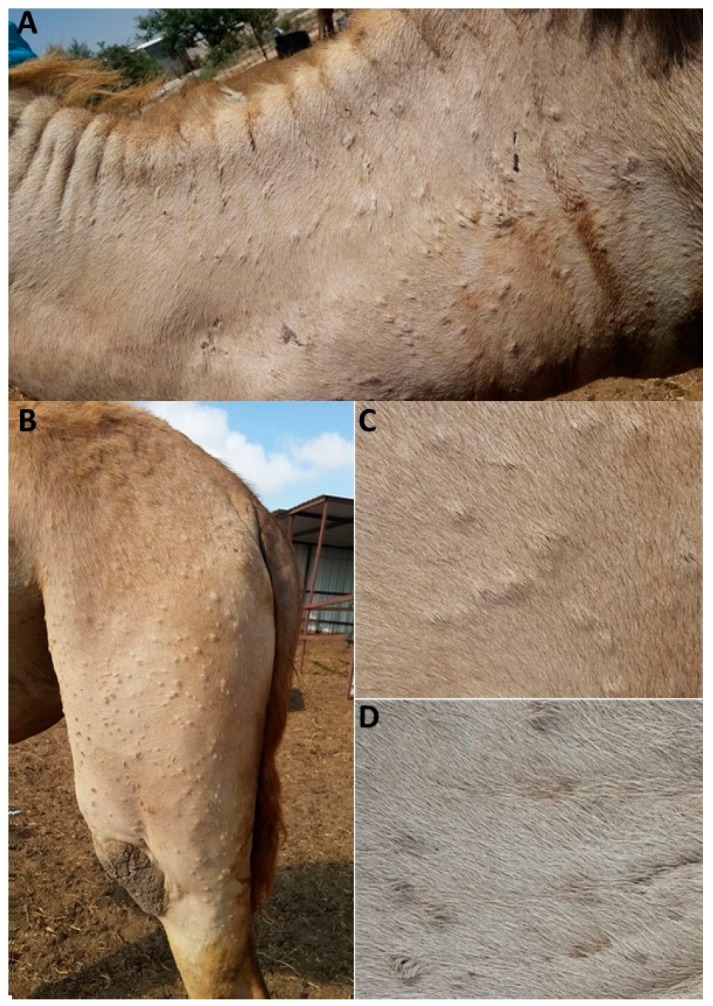
Skin lesions observed in infected camels. (**A**) Skin lesions on the neck and (**B**) hip of the left rear leg of an infected camel. Enlargement of (**C**) stiff papules and (**D**) ulcerated papules with purulent secretion.

In the summer of 2016, a disease with skin manifestation was reported in camels in the southern part of Israel. Infected animals were reported in two herds in the Be′er-Sheba district, one in the Tel-Arad region, and one in the Hebron region. Inspected herds of mainly adult females exhibited symptoms including weakness, loss of appetite, fever, abortions, and multifocal lesions on the skin (Figure 1A–D). During the initial stages of the disease, stiff papules were observed, while at the advanced stage they become uncerated with purulent secretion. No mortalities were reported, and the inspected animals recovered after a few weeks. There were no subsequent reports of further spreading of the disease. No infections in husbandry personnel were reported. In order to identify the etiologic agent, skin samples from eight representative camels were subjected to various diagnostic tests.

### 3.2. Diagnostic Transmission Electron Microscopy

**Figure 2:**
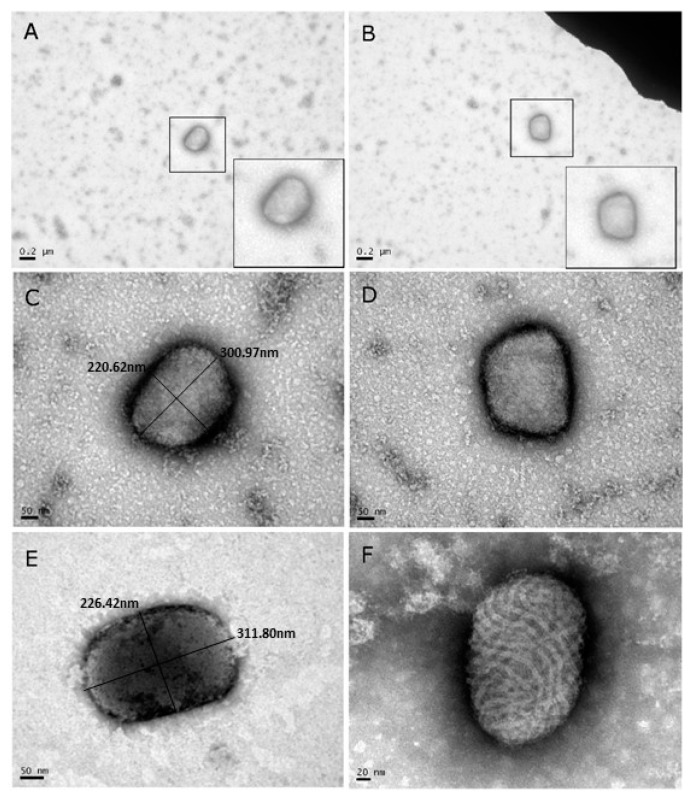
(**A**–**E**) Transmission electron microscopy (TEM) images of viral particles extracted from lesions taken from four individual camels and negatively stained with 1% Phosphotungstic acid (PTA). Note that the morphology and dimensions of the virus particles corresponds with the morphology and dimensions of the *Orthopoxvirus* (OPV) family. (**F**) For comparison, a TEM image of an orf virus after negative staining. Note the profound morphology differences between the orf virus (**F**) that belongs to the PPV family, and the OPV family (**A**–**E**).

Samples from camel skin lesions were prepared for diagnostic TEM. Out of the eight samples, four were analyzed by TEM. All four negatively-stained samples clearly showed the characteristic brick-shaped morphology of OPV (Figure 2A–E), in contrast to the oval shape of PPV with tubules in a crisscross pattern (Figure 2F). At least 20 particles could be identified in one grid. The dimensions of the viral diameter were 217.6 ± 18.7 × 293.15 ± 18.8 nm. These values are in accordance with those reported in the literature [23]. Thus, diagnostic TEM indicated the existence of OPV-like virus in the skin of camels. No papillomavirus particles were observed.

### 3.3. Pock Formation on CAM

**Figure 3:**
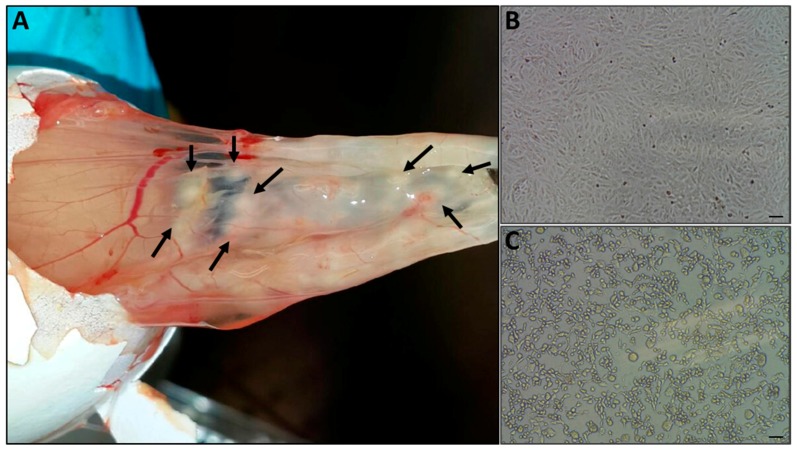
Virus isolation. (**A**) Infected skin samples extracts were used to infect chorioallantoic membrane of embryonated chicken eggs, and the onset of pock lesions was observed four days post-injection (marked with arrows). The pocks were excised from the membrane, and used to generate a suspended inoculum for the infection of Vero cells. Cytopathic effect was observed four days after inoculation (**C**), while the uninfected cells maintained normal morphology (**B**), Bar =100 μm.

The chorioallantoic membranes (CAM) of 10-day old embryonic chicken eggs were infected with skin extracts, as described above. Egg death occurred four days post-injection, at which time white pocks were observed on the membrane (Figure 3A). The pocks were excised from the membrane, and resuspended in sterile PBS.

### 3.4. CPE on Vero Cells

The resuspended pock material was used to infect Vero cells. Cytopathic effects (CPE) were observed four days post-infection (Figure 3C). Virus titer was determined as TCID50 10^5^/mL.

### 3.5. Immunofluorescence Assay

**Figure 4:**
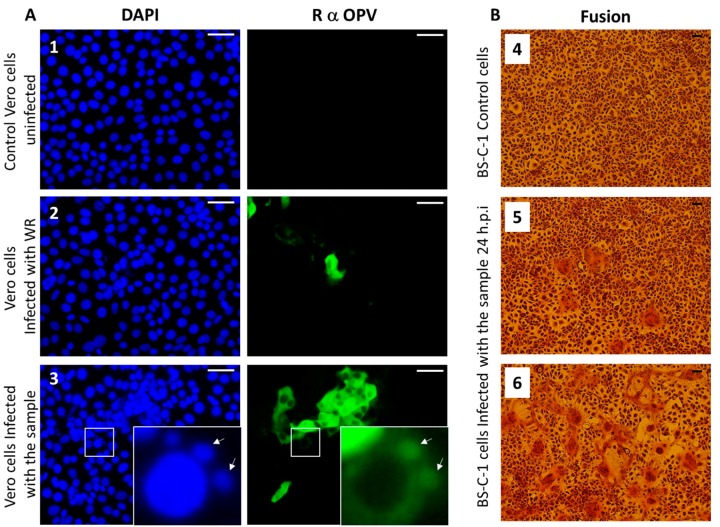
Immunofluorescence and syncytia formation assays. (**A**) Vero cells were infected with (**2**) Vaccinia virus-Western Reserve (VACV-WR) at a multiplicity of infection (MOI) = 0.01, or (**3**) skin lesion suspensions or (**1**) left uninfected. At 24 hours post-infection, cells were fixed and stained for Nuclei (DAPI: blue) and *Orthopovirus* antigens (anti-VACV: green). Arrows indicate viral factories. (**B**) Induction of cell–cell fusion (syncytia) by camelpoxvirus CMLV is infection titer-dependent. BS-C-1 cells were infected at different dilutions. (**4**) Control uninfected BS-C-1 cells (**5**) BS-C-1 cells infected at MOI = 0.005 and (**6**) at MOI = 0.05. Bar = 100 μm.

Members of the OPV family share common antigens [23]; thus, the use of OPV-specific antibodies generated against VACV, the prototype of OPV, can recognize other members of the OPV family. We performed an immunofluorescence assay using anti-OPV hyperimmune serum on Vero cells infected with the suspended pock material, as described in Section 2.4. Our results show infected cells that were positively labeled by the anti OPV hyperimmune serum (Figure 4A).

Moreover, viral factories stained by DAPI and anti-OPV antibodies, which are characteristic of OPV-infected cells, can be seen in the infected cells (Figure 4A-3). These results support the hypothesis that the infectious agent belongs to the OPV family. As this sample was obtained from a camel, and taking into consideration that CMLV is a species-specific OPV, these findings support our assumption that the OPV isolated from the camels dermal lesions contains CMLV.

### 3.6. Syncytia Formation in Cultured Cells

Syncytia (cell–cell fusion) formation is characteristic of several pathogenic OPV members, including CMLV, ectromelia virus (ECTV), and monkeypox (MPXV) [24]. In order to verify the ability of the isolated virus to induce cell–cell fusion, BS-C-1 cells were infected with the sample at multiplicities of infection (MOI) of 0.005 and 0.05 (Figure 4B, panels 5 and 6, respectively). Cells were incubated for 24 h, and cytopathic effects were evaluated. The most prominent cytopathic effect was the formation of syncytia, which was manifested by polykaryocytosis (Figure 4B). These giant multinucleated cells comprised few to dozens of nuclei, which were positioned in many cases in a ring-shape form. These results further supported the assumption that the infectious agent is CMLV.

### 3.7. CMLV Identification Using PCR

In order to enable a definitive identification of the etiological agent of the disease, PCR was employed, with both rapid SYBR-based qPCR, and the amplification of several genomically distant regions for sequencing. For preliminary detection and identification, two separate qPCR were used, identifying different regions in the CMLV genome. The C18L test [22] and the “137,637” test were developed in this study. The rationale for developing an additional test was to confirm the results obtained using the C18L test, by targeting a different region in the CMLV genome. The selected target region is located downstream to the gene, encoding the RNA-dependent DNA polymerase subunit B (reference accession No. NC_003391). This region is non-coding, and its variability among different OPV species is relatively high (data not shown), thus enabling the design of a CMLV-specific reaction. By using this region as the target, the specificity of the sample analysis was further increased, thereby further minimizing the chance of erroneous identification. Additionally, in all of the annotated CMLV sequences, there were two mismatches in the reverse primer of the C18L reaction [22]. In order to maximize the chances of a successful amplification, we developed the “137,637” test, consisting of primers with 100% complementarity to the annotated sequences.

**Figure 5:**
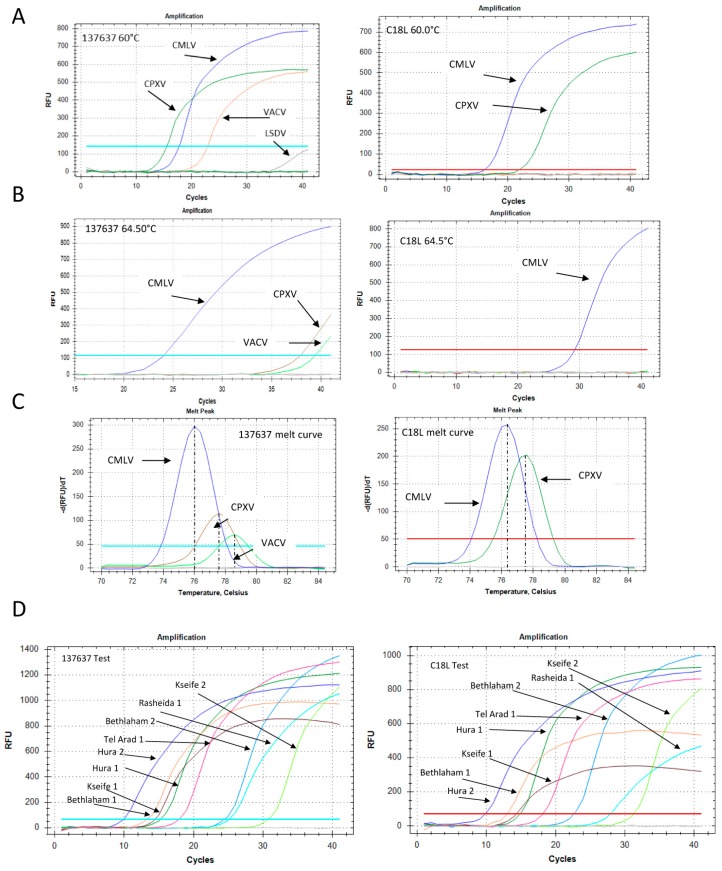
Development of *Orthopoxvirus*-specific qPCR and samples examination. (**A**) qPCR of Camelpoxvirus (CMLV), cowpox virus (CPXV) and Vaccinia virus (VACV) at 60 °C annealing temperature. Left panel: The “137,637” reaction. Right panel: The C18L reaction. (**B**) Similar reactions as in (**A**), but with an annealing temperature of 64.5 °C. (**C**) Melt profiles of the “137,637” (left) and C18L (right) reactions at 60 °C annealing temperature. (**D**) Representative results of the “137,367” (left) and C18L (right) tests that were used to detect and confirm the presence of the virus in eight samples from four locations, as detailed in Section 3.1. Blood and skin DNA extracts were both positive. The C_t_ values for each sample are detailed in Table S1.

In order to determine the sensitivity of the new qPCR, we performed a calibration test to compare its sensitivity with that of the C18L test (Figure S1). The two tests showed similar performances, detecting less than 10 target copies. The new test showed a slightly higher efficiency compared with the C18L test: 90.9% versus 82.7%, respectively (Figure S1). The specificity of the two qPCR was examined by using different poxvirus samples as template. DNA extracted from the orf virus (PPV) and lumpy skin disease virus (capripoxvirus) did not yield any product (data not shown). The primers designed for the “137,637” reaction were identical to the CMLV-corresponding genomic region (accession NC_003391), but contained two and three mismatches with respect to the corresponding VACV and CPXV virus regions (accession numbers LN864565 and AY313848, respectively). However, under permissive conditions, i.e., 60 °C annealing temperature, DNA samples of these two OPV were positive (Figure 5A).

The specificity of the reaction increased significantly following an increase of the annealing temperature to 64.5 °C, at which point the CMLV signal remained robust, while the CPXV and VACV signals decreased markedly (Figure 5B). Moreover, the melt profile, determined by the post-amplification melt analysis, was different for each sample, thus enabling specific identification of the CMLV virus even under permissive reaction conditions (Figure 5C). The C18L test detected both CMLV and CPXV, but not VACV, in the 60 °C annealing reaction, with different melt profiles (Figure 5A,C), and only CMLV in the 64.5 °C annealing reaction (Figure 5B). A total of eight samples (skin and blood) obtained from four locations (Section 2.2) were identified (Figure 5D). Both the C18L and “137,637” tests gave similar results, with C_t_ values ranging from 9.87 to 31 (Table S1).

**Figure 6:**
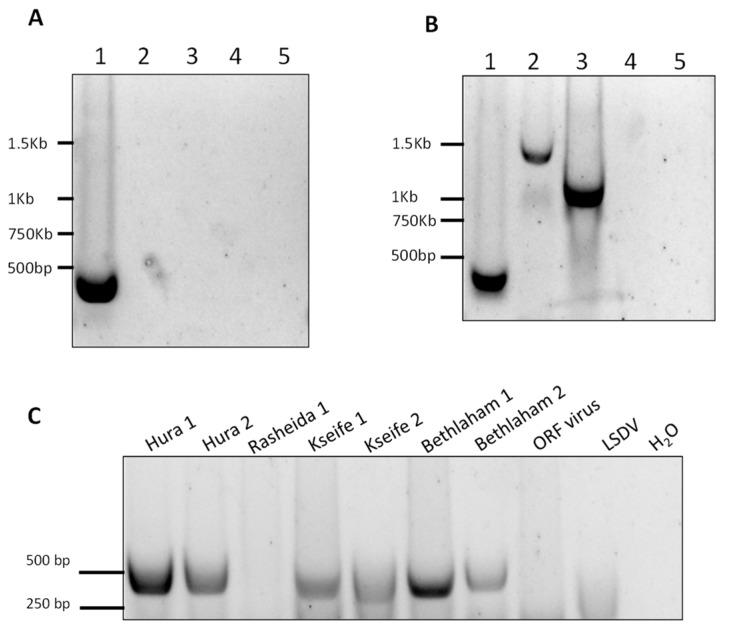
Development of standard PCR for differential detection of camelpoxvirus (CMLV), cowpoxvirus (CPXV), andvaccinia virus (VACV) (**A**,**B**) and samples examination (**C**). (**A**,**B**) Lane 1 = CMLV, lane 2 = CPXV, lane 3 = VACV. As negative controls, lumpy skin disease virus (capripox, lane 4), and orf virus (parapox, lane 5) were used. (**A**) PCR for the amplification of the region spanning positions 15,458 to 15,877 with an extension time of 20 s allowed the amplification of a 420-bp length product from CMLV only (lane 1). (**B**) Increased extension time of one minute and 20 s enabled the amplification of CMLV, CPXV, and VACV DNA samples with different product lengths. (**C**) A 420-bp product was detected in seven out of the eight samples, and was used as an additional test for the quantitative analysis and sequencing of a CMLV-specific amplified region.

In order to further establish the identity of the isolated virus, we developed three standard PCR to amplify three regions within the CMLV genomes. The first reaction was designed to amplify the region between positions 15,458 and 15,877 in the CMLV genome (accession NC_003391). This reaction specifically identified CMLV and distinguished it from other OPV, namely CPXV and VACV. This was accomplished by amplifying a region whose length is 420 bp in the CMLV genome, 1140 bp in the CPXV genome (accession LN864565), and 1048 bp in the VACV (accession AY313848). By keeping the extension time of the reaction below 25 s, only the CMLV presence could be detected (Figure 6A).

**Figure 7:**
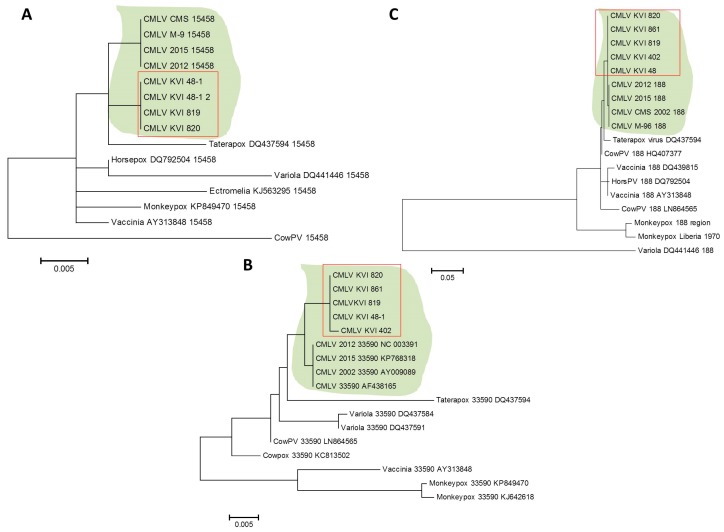
Phylogenetic analysis of isolated camelpoxvirus (CMLV) samples. The sequences of three different (not consecutive) regions in the CMLV genome were compared to annotated sequences of CMLV and related *Orthopoxviruses* (OPV). Analysis was performed following multiple alignments, as described in Section 2.6. (**A**) Analysis of the region spanning positions 15,458 to 15,877 in sequence number NC_003391 (420 bp size). (**B**) Analysis of the region spanning positions 33,590 to 34,492 in sequence number NC_003391 (902 bp size). (**C**) Analysis of the region spanning positions 188,946 to 190,040 in sequence number NC_003391 (1094 bp size). The CMLV clade is shaded, and the KVI (Kimron Veterinary Institute)-isolated samples are marked with a rectangle.

However, by extending this stage to one minute and 25 s, two longer products were generated, demonstrating that under permissive conditions, this test could differentially detect CMLV and other related viruses (Figure 6B). This reaction successfully amplified the target sequence in all of the samples, except one sample, which could not be amplified despite repeated attempts (Figure 6C). Two other regions within the CMLV genome were amplified and sequenced, one between positions 33,590 and 34,492 in sequence number NC_003391 (902 bp), and the other between positions 188,946 and 190,040 in sequence number NC_003391 (1094 bp). The sequences were deposited in the GeneBank with the following accession numbers: KX770279–KX770292. Multiple alignments followed by phylogenetic analyses showed that all of the examined samples clustered to the same clade, thereby generating a distinct clade, separated from the other annotated CMLV isolates (Figure 7).

The sequenced samples shared a high degree of identity, and were different from the annotated CMLV isolates available from GenBank (Table S1). Collectively, these results suggest that all of the CMLV samples identified in this study belong to the same subtype, which is different from the annotated CMLV isolates currently available from the NCBI GenBank.

## 4. Discussion

This study reports the first documented outbreak of camelpox disease in Israel, and demonstrates the diagnostic procedures used for its diagnosis, which include morphological, cellular, immunological, and molecular methods. The employment of several approaches based on different features of the causative agent significantly improved the reliability of the diagnosis [25]. Since then, no other CMLV cases in this area were reported (International Society for Infectious Diseases–ProMED).

The disease occurs mainly in adult females camels, and is manifested by skin lesions on hairless skin regions such as the face and neck, whereas in several cases, lesions appear throughout the body. Symptoms such as fever, weakness, lack of appetite, and abortion were observed with no mortality. Since camels serve as transport and milk-producing animals, such a disease that is infectious and easily transmitted will have profound economic implications on societies that rely on camels.

Eight skin and blood samples from sick camels were examined in this study. A disease involving exanthematous skin conditions in camels raises several possibilities regarding the causative agent: camelus dromedary papillomavirus, CCEV, CMLV, or co-infection with CCEV and CMLV. This necessitated the use of a differential diagnosis using several methods, the first of which was diagnostic TEM [26]. It should be noted that this approach, which was performed on negatively stained samples, is fast and relatively simple, but requires expensive and complicated equipment, as well as professional expertise. Indeed, in the current study, using TEM, the viral particles could be observed easily with their characteristic brick-shape morphology, assigning them to the OPV family. For comparison, the CCEV, which causes a disease with similar symptoms, belongs to the PPV with a different morphology, which is characterized by an oval shape and a tubules’ texture with crisscross patterns. Hence, TEM is a powerful technique for differentiation between OPV and PPV [27].

Further examination and characterization was carried by the infection of CAM and Vero cells. While the infection of SPF-ECE resulted in typical pocks formation, the infected Vero cells were positive by immunofluorescence using an OPV-specific antibody. In order to determine the virus identity at the species level, PCR followed by sequence analysis was used. Of the various OPV, the primary candidate was CMLV. To establish this, we employed the C18L test [22], and due to sequence variation in the region amplified in the C18L test, as evident from CMLV genomes deposited recently in GenBank, an additional test was developed. The new PCR developed in this study was based on a non-coding genome sequence that shows significant variation among the OPV family, thus allowing the specific identification of CMLV. Using this test in parallel with the C18L PCR, we clearly demonstrated that the infecting virus is CMLV.

The recent CMLV-annotated genomes suggested the presence of a different CMLV. The three endpoint PCR tests aimed to establish the subspecies identity of the isolated virus, and showed that all of the tested samples were of the same subspecies, which was distinct from the other four annotated subtypes currently available from GenBank. The regions selected for the analysis contained variations and insertions that enabled a clear distinction between CMLV and other OPV species, such as monkeypox, cowpox, and rabbitpox viruses (Figure 7, Table S2).

Recent studies published after the completion of this manuscript compared a large number of samples using the HA and B2L genes [28] and the C18L gene [13], which were not studied here. The completion of the full genome sequence of the newly isolated subtype (work in progress) will allow for a better comparison, and will also determine its similarity to other studied isolates. Nonetheless, this report describes the first identification of CMLV in Israel, and highlights the significance of a combined approach for the quick and definitive identification of a pathogen that had been previously unreported in this country. Such a prompt response is of major importance in order to launch effective treatment and prevention processes. Moreover, we describe new PCR tests that can (1) distinguish between CMLV and related OPV species (VACV, CPXV) that exhibit broad species tropism, and (2) determine that the new, CMLV isolate is genetically distinct from the currently annotated camelpox isolates.

## 5. Conclusions

This study describes the first diagnosed outbreak of camelpox virus in Israel. Although camelpox virus infects camels, it is genetically the closest relative of the variola virus, the causative agent of smallpox. Although rare, human infections of camelpox have been previously described. The findings in this study adds to the recent emergence of *orthopoxvirus* human infections, raising the need for adequate diagnostic approaches, as outlined in this study.

## Figures and Tables

**Table 1:** Primer sets used for characterization of the infecting camelpox virus (CMLV).

Primer Name	Primer Sequence	Amplicon Length in Sequence No. NC-003391
15458 Fwd	5′-CTATATCTATATGAGATGAC-3′	420 bp
15877 Rev	5′-GTTGGTAGTAGGGTACTCGTG-3′	
CMLV 33590 F	5′-TCTGGAAGTGGATATACATAG-3′	902 bp
CMLV 34492 R	5′-GGGATAATCCAGAATTGATAATAGTGG-3′	
CMLV 188946 F	5′-GTATAATGTATGTAACCCGTAC-3′	1094 bp
CMLV 190040 R	5′-TACATACCATTAATAATGCAAGC-3′	

## Supplementary Materials

The following are available online at http://www.mdpi.com/1999-4915/10/2/78/s1.
