# Supplementary material for: First Diagnosed Case of Camelpox Virus in Israel
Source: Viruses. 2018 Feb 13;10(2):78. doi: 10.3390/v10020078 (PMC5850385; doi:10.3390/v10020078)
Supplement: Supplementary file 1 [file viruses-10-00078-s001.docx]

**Table S1.** Ct values for representative CMLV samples as detected in the 137637 and C18L tests.

| **Sample** | **Ct**  **137637 test** | **Ct**  **C18L test** |
| --- | --- | --- |
| Hura 1 | 15.18 | 14.9 |
| Hura 2 | 9.87 | 9.87 |
| Bethlaham 1 | 13.26 | 13.06 |
| Bethlaham 2 | 24.34 | 22.65 |
| Rasheida 1 | 25.07 | 28.01 |
| Kseife 1 | 14.36 | 14.35 |
| Kseife 2 | 30.69 | 31.28 |
| Tel Arad 1 | 17.84 | 18.16 |
| H_2_O | N/A | N/A |

**Table S2.** Similarity values of CamPV amplified regions. The alignment was used to construct the phylogenetic dendrograms in Figure 7. Alignment was performed using the ClustalW algorithm embedded in the Geneious software. Similarity calculations were performed using the Geneious software.

| Region in sequence no. NC_003391 | Comparison | Similarity (%) |
| --- | --- | --- |
| 15458 - 15877 | New isolates^1^ | 100 |
| 15458 - 15877 | New isolates Vs. annotated CamPV sequences^2^ | 99.5 |
| 15458 - 15877 | Overall Orthopox viruses^3^ | 91.0 |
| 33590 - 344492 | New isolates^3^ | 99.9 |
| 33590 - 344492 | New isolates Vs. annotated CamPV sequences^2^ | 94.8 |
| 33590 - 344492 | Overall Orthopox viruses | 88.3 |
| 188946 - 190040 | New isolates^3^ | 99.7 |
| 188946 - 190040 | New isolates Vs. annotated CamPV sequences^2^ | 97.9 |
| 188946 - 190040 | Overall Orthopox viruses | 75.6 |

^1^Similarity among sequences of four isolates obtained in this study

^2^Similarity among Camelpox sequences obtained in this study and four GenBank-annotated Camelpox sequences

^3^Similarity among sequences of five isolates obtained in this study

| **** |
| --- |

**Figure S1. Calibration of the C18L and 137637 tests.** Both tests were examined for sensitivity and efficiency using serial dilutions of a purified PCR product at calculated concentrations. The efficiency of the 137637 reaction (top) was slightly higher than that of the C18L reaction. Both tests successfully detected less than 10 target copies.
